# Antiretroviral drug resistance and viral tropism in HIV-1 CRF06_cpx infected patients failing antiretroviral (ARV) therapy

**DOI:** 10.1186/1471-2334-14-S4-O22

**Published:** 2014-05-29

**Authors:** Merit Pauskar, Kristi Huik, Radko Avi, Eveli Kallas, Ene-Ly Jõgeda, Tõnis Karki, Svetlana Semjonova, Jelena Smidt, Lilia Novikova, Külliki Ainsalu, Irja Lutsar

**Affiliations:** 1 Department of Microbiology, University of Tartu, Tartu, Estonia

## 

While the prevalence of X4-tropic viruses in subtype B is relatively well studied the distribution of tropism in CRF06_cpx (causing Estonian epidemic) is less known, especially in the treatment experienced patients with established multidrug-resistance and who are candidates for therapy with CCR5 antagonist.

Aim: to describe HIV-1 drug resistance mutations (DRMs) and co-receptor tropism in antiretroviral (ARV) treatment failing patients infected with HIV-1 CRF06_cpx and to establish their suitability to treatment with CCR5 receptor antagonists.

Altogether 12 patients were studied – all with ARV therapy failure and considered to start CCR5 antagonist therapy. Genomic viral DNA was directly sequenced in pol region and V3 loop of gp120 in triplicates. HIV-1 DRMs and tropism were detected using Stanford University Drug Resistance Database and Geno2pheno_[coreceptor]_ 2.5. algorithm, respectively. All sequence prediction results with a false-positivity rate above 10% were considered R5-tropic.

Of 12 viruses 11 were successfully sequenced and analyzed. Patients have been diagnosed and ARV therapy initiated in 2002-2013. 8/11 cases had failure of the second ARV regimen, 4/11 of the third and in 2/11 of the fourth ARV regimen. Median viral load at the time of tropism testing was 53,326 (IQR 16,328-113,018) and median CD4+ count 184 (IQR 142-217) cells/cmm. The most commonly used regimens were EFV + 3TC plus AZT (5 cases) or plus ABC (4 cases). Of 11 viruses 10 had resistance against two ARV classes (NRTI + NNRTI or NNRTI + INI) and 1 had triple class resistance (NRTI + NNRTI + PI). In NRTI treated population the most common primary DRM was M184V (10/11), followed by L74V/IL/I and K70E/R/EKQ (both 4/11). In NNRTI treated population DRMs were as follows: K103N (5/11), P225H (4/411), G190A/S and K101H/E (both 3/11), others were seen in lower frequency. In PI treated patients two primary DRMs were represented – I54V, V82A (both in same case). In INI treated population one primary DRM was represented – Y143R. The majority of viruses (10/11) were R5-tropic and all determined as CRF06_cpx by phylogenetic analysis.

The Estonian HIV epidemic has reached the stage where the first patients are considered to start treatment with CCR5 antagonists. Almost all highly resistant viruses were R5-tropic suggesting that CCR5 antagonists are an appropriate option for CRF06_cpx viruses resistant to other ARV agents.

